# Tenotomy versus Tenodesis in the treatment of the long head of biceps brachii tendon lesions

**DOI:** 10.1186/1471-2474-13-205

**Published:** 2012-10-22

**Authors:** Olimpio Galasso, Giorgio Gasparini, Massimo De Benedetto, Filippo Familiari, Roberto Castricini

**Affiliations:** 1 Department of Orthopedic and Trauma Surgery, School of Medicine, University “Magna Græcia” of Catanzaro, Viale Europa (Loc. Germaneto), 88100, Catanzaro, Italy; 2 Department of Orthopedic Surgery, Villa Verde, Piazzale Kennedy, 63900, Fermo, Italy

**Keywords:** Long head of the biceps brachii tendon, Tenotomy, Tenodesis, Shoulder, Outcomes

## Abstract

**Background:**

The superiority of tenotomy vs. tenodesis for surgery on lesions of the long head of the biceps brachii tendon is still under debate. Indeed, high-quality evidence is lacking, mainly because of methodological problems, such as retrospective design, population sample size or lack of patient randomization.

**Methods/Design:**

The study will be a two-center, double-blind, randomized, controlled trial to compare patients treated with biceps tenotomy or tenodesis for lesions of the long head of the biceps brachii tendon over a 2-year follow-up period. The study participants will be 128 adults with biceps brachii tendinopathy and supraspinatus tendon tears. The primary end point will be the postoperative difference in the Constant-Murley score (CMS) between the 2 groups at the two-year follow-up. A comparison of the mean improvement with standard age- and gender-related CMS will be performed. The secondary end point will be evaluation of the postoperative general health of patients, as evaluated with Short Form 36 (SF-36) scores. The number and severity of complications associated with use of the different surgical techniques will be assessed.

**Discussion:**

This study will be the first randomized and appropriately powered clinical trial to directly compare tenotomy and biceps tenodesis. The results of this study will help to establish clinical practice guidelines for patients suffering from lesions of the long head of the biceps brachii tendon, providing important information to patients and health care providers about the possible complications, outcome predictors and effectiveness of the targeted interventions.

**Trial Registration:**

Current Controlled Trials ISRCTN38839558

## Background

Lesions of the long head of the biceps brachii tendon (LHBT) are common [1,2], and when not adequately treated, they may be responsible for persistent pain, as well as functional impairment of the shoulder [3]. LHBT lesions can be isolated [4] but are more frequently associated with more complex disorders, such as shoulder instability or supraspinatus tendon tears [5-7]. The decision about whether to proceed with conservative [8-12] or surgical management of LHBT lesions might depend on the associated shoulder disorders and the duration of the symptoms [13].

The two most common procedures for LHBT lesions are biceps tenotomy and biceps tenodesis [14]. Currently, there is no consensus regarding the most effective surgical procedure because of the inconsistent results and the limitations of the published studies [15,16]. Some authors have claimed that tenotomy is superior and reported satisfactory results in most patients treated with this technique [17-19]. Tenotomy has the advantages of easier execution and, when performed alone, the requirement of fewer restrictions during the postoperative period with an earlier return to activity [15]. Other authors reported similarly good results in most patients treated with tenodesis of the LHB [20-23]. This technique seems to be able to prevent some of the most common complications associated with tenotomy, such as cramping of the brachial biceps muscle and retraction of the biceps tendon [18,24]. The latter complication, referred to as Popeye’s sign or Popeye’s deformity, occurs in 3-70% of patients undergoing tenotomy of the LHBT and results in a cosmetic deformity [17,20]. Further possible complications related to the intervention represented by tenotomy include decreases in the strength of flexion and supination of the arm and forearm, respectively [11,25]. However, these findings are not consistent with the results reported by others [16,24,26].

Most of the studies that compare the results of LHB tenodesis and tenotomy are limited by methodological deficiencies, such as retrospective design, low statistical power or lack of patient randomization [14]. To the best of our knowledge, there are no double-blind randomized controlled trials on this topic. The purpose of this double-blind randomized trial is to compare the effectiveness of tenodesis and tenotomy in the treatment of LHBT lesions.

## Methods/Design

This will be a two-center, double-blind, randomized, prospective study of all consecutive patients to be treated for LHBT lesions with biceps tenotomy or biceps tenodesis, including a 2-year follow-up period.

This study will require 12 months for patient enrollment and an additional 2 years for the completion of follow-up over a total of 3 years.

### Aims and hypothesis

This study will seek to compare the efficacy of biceps tenotomy and biceps tenodesis in the treatment of LHBT lesions. The primary end point will be the postoperative differences in Constant-Murley scores (CMS) between the 2 groups at the two-year follow-up. The secondary end point will be evaluation of the general health of patients in both groups as evaluated by Short Form 36 (SF-36) scores. The number and severity of complications associated with the investigational surgical techniques will be assessed. The null hypothesis is that there will be no difference in the mean CMS between the tenotomy and tenodesis groups.

### Inclusion and exclusion criteria

A screening interview will be conducted to ensure that the referred patients meet the inclusion/exclusion criteria prior to performing study-specific tests. A thorough physical examination and an MRI of the shoulder will be performed for identification and characterization of the pathology. The diagnosis will be verified by arthroscopy.

Male or female patients, aged 40 years or older, with LHBT lesions (tenosynovitis, subluxation, dislocation or partial rupture of the tendon) associated with grade I or II full-thickness supraspinatus tendon tears [27] will meet the criteria for enrollment. If any of the following criteria apply, patients will be excluded: (1) previous surgery of the affected shoulder, (2) insufficient comprehension of the Italian language to understand the trial features, (3) mental handicap, (4) a lack of willingness to return for all scheduled follow-up visits, (5) participation in another study, (6) any previous upper extremity neurological disorder or diagnosis based upon physical examination, (7) complaint of pain in both shoulders, (8) a life expectancy of less than 2 years, (9) an ongoing insurance trial, lawsuit, or pending legal action for shoulder disease.

Patients who do not meet all of the inclusion criteria and patients who meet any of the exclusion criteria will automatically be excluded from the study. Informed consent will be obtained from each patient.

### The number and source of patients

Patients will be recruited from the outpatient clinic. The goal is to assess 128 participants for the study. We performed a priori power analysis to determine the sample size required to achieve the pre-specified power level (1 - β), significance level α, and the population effect size for the CMS between the two treatment groups to be assessed. In a previous study, the CMS was normally distributed within some subject groups [28]; therefore, we will use an unpaired t-test with an associated medium effect size equal to 0.5. Under these conditions, we will need to study 64 subjects for each group to be able to reject the null hypothesis that the population means of the groups are equal with a probability (power) of 0.8 and that the Type I error probability is equal to 0.05. Considering the same significance and power level, this sample size will allow us to detect an effect size that is equal to 0.35 for the null hypothesis that the population means of CMS scores before and after treatment are equal. Because of the possibility that participants could drop out of the protocol, a total of 150 patients will be recruited for the trial. G*Power software (Institut fur Experimentelle Psychologie, Heinrich Heine Universitat, Dusseldorf, Germany) was used for the power analyses.

### Randomization

After the participants have gone through the screening process and are determined to meet the enrollment criteria, they will be randomized to the treatment groups (i.e., biceps tenotomy or biceps tenodesis). The patients will be randomly allocated to the groups using specific software (Filemaker Pro 12, Santa Clara, CA, USA) with an allocation ratio of 1:1; they will be unaware of which surgical treatment they will receive. Patients will be fully informed that they have a 50% chance of receiving LHB tenotomy and that there will be no opportunity for cross-over treatments.

An independent secretary will organize treatment allocation by distributing sealed numbered envelopes to the nurse manager in the operation theatre. A nurse will open the envelope only when a preoperative diagnostic evaluation has documented the LHBT lesion and supraspinatus tendon tear.

### Imaging

Preoperative radiological examination will include anterior-posterior (AP) and outlet X-ray and a 1.5-T non-arthrographic MRI of the shoulder (Magnetom® Symphony-Maestro-Class, Siemens AG, Erlangen, Germany). Indeed, non-contrast MRI has been reported to be 98% sensitive and 89.5% specific for biceps tendon pathology [29]. Imaging scans will include a fast spin-echo intermediate-weighted axial sequence, a fast spin-echo coronal oblique intermediate-weighted sequence, and coronal oblique and sagittal oblique fast spin-echo T2-weighted acquisitions with fat suppression. A full-thickness tear will be defined as a high T2 signal extending through the depth of the supraspinatus tendon [30].

Reports will be generated by two musculoskeletal radiologists who will be blinded to the clinical characteristics of the patients, and their evaluation will be repeated on two separate days. Cohen’s kappa coefficient for the inter-observer and intra-observer reliability of scoring will be calculated. A consensus decision on the presence of supraspinatus tendon tear and LHBT lesion (i.e., tenosynovitis, subluxation, dislocation or partial rupture) will be reached in a final common readout.

### Intervention

The investigator performing the surgical intervention will be blinded to the patient’s baseline and follow-up clinical measurements. The surgeon will know which of the two techniques will be employed for each patient during the surgical procedure. One experienced shoulder surgeon (RC) will perform all operations.

Both procedures will be performed in the lateral decubitus position. The same number of skin incisions will be required for the arthroscopic portals to repair the supraspinatus tear and the LHB tenotomy or tenodesis. A routine glenohumeral diagnostic arthroscopy will be performed through a standard posterior arthroscopic portal; lateral and rotator interval anterior portals will be used to repair the supraspinatus tendon tear with a single row technique after biceps treatment. Twinfix^TM^ Ti 5.5 mm suture anchor with three #2 Ultrabarid^TM^ sutures (Smith & Nephew Inc, Memphis, TN, USA) will be used to repair the supraspinatus tendon.

### Biceps Tenotomy

Tenotomies will be performed through the anterior glenohumeral portal. A blunt probe will be used to pull the extra-articular portion of the biceps into the glenohumeral joint to allow for visual inspection of the macroscopic structural changes [31]. The biceps tendon will then be released from its insertion on the superior glenoid labrum using arthroscopic electrocautery.

### Biceps Tenodesis

A biceptor^TM^ tenodesis system (Smith & Nephew Inc, Memphis, TN, USA) will be used during the procedure. A spinal needle will be inserted at the anterior-lateral edge of the acromion into the shoulder joint and through the biceps. A monofilament suture will be introduced through the needle and the biceps tendon. Then, the long head of the biceps tendon will be cut at its insertion point through the rotator interval portal under the glenoid labrum. Both arms of the suture will exit the anterior portal. The biceps tendon will be mobilized out of the bicipital groove, and the desired position for reattaching the tendon will be located. A 2.4-mm guide wire will be drilled perpendicularly to the humeral shaft in the bicipital groove. A reamer appropriate to the screw diameter will be used to drill a 25-30-mm deep hole for definitive tendon attachment.

A tendon fork will be placed after tension release of the tendon, and the tendon will be inserted into the prepared hole up to the far cortex. The tendon will subsequently be pinned in place and a biosure peek-optima® (Smith & Nephew Inc, Memphis, TN, USA) interference screw will be inserted over this pin. The excess tendon will be trimmed.

### Postoperative rehabilitation

The operated shoulder will be immobilized for 3 weeks using a sling with an abduction pillow. Pendulum exercises will be allowed, starting from the first postoperative day. After the immobilization period, passive and assisted exercises in forward flexion and external rotation will be initiated. Strengthening exercises will be restricted until 6 weeks after the surgical procedure. Three months after the operation, patients will be allowed to engage in light sports activity. Heavy manual work and overhead motion will be allowed after 6 months.

### Baseline and follow-up measurements

The baseline measurements and 1, 6, and 24-month follow-up outcomes will be evaluated by two trained attending physicians (MD and FF) blinded to the patient’s treatment assignment. Demographic data, such as age, gender, dominant arm, activity level (1 = sedentary, 2 = light manual work, 3 = heavy manual work), concomitant injuries, surgical side, disease onset (0 = acute, 1 = insidious), symptom duration, and prior failed conservative therapies, will be collected at the beginning of the study.

The following shoulder tests will be performed at baseline and at the follow-up examination: Speed’s test [32], Yergason’s test [33], the palm-up test [34], the active compression test [35], Neer’s sign [36], Jobe’s test [37], and the lift-off test [38].

Physical examination will include bilateral measurement of the active range of motion of the shoulder, elbow flexion and extension and forearm pronosupination with a goniometer. Shoulder motions will include forward flexion, abduction, external rotation and internal rotation at 0° of abduction. The latter measurements will be carried out with the patient in a sitting position to prevent hyperlordosis. Scapular rotation will be restricted during abduction and forward flexion by the examiner’s hand.

CMS and SF-36 scores will be measured preoperatively and at follow-up. The CMS was introduced to determine the functional outcome after treatment for a shoulder injury [39,40]. This score (0–100) is divided into four subscales, including pain (15 points maximum), daily living activities (20 points maximum), range of motion (40 points maximum), and strength (25 points maximum). The higher the CMS score, the better the shoulder’s condition. The postoperative difference in the CMS between the groups will be evaluated at the two-year follow-up. Comparison of these outcomes with standard age- and gender-related CMS will be performed. As previously described, patients will be considered a treatment success if their CMS is at least 80% of the standard age- and gender-related value at the study’s endpoint [27,41].

The baseline and follow-up isometric muscle strength will be evaluated with the 500 N Mecmesin Myometer (Mecmesin Co, Nottingham, UK) as previously indicated for shoulder [42,43] and elbow [44] strength measurements. External shoulder rotation will be tested with the shoulder in a neutral position and the elbow in 90° of flexion. The strap of the myometer will be applied to the distal forearm of the patient. When measuring shoulder strength during external rotation with the arm at the side, the subject will be asked to externally rotate only the forearm with the elbow kept at the side to prevent compensation from motion of the torso or extension-abduction of the humerus. Abduction shoulder strength will be tested with the patient in the standing position and the arm in 90° of abduction in the scapular plane with the elbow extended and the forearm pronated, and resistance will be applied to the wrist. If 90° of abduction cannot be achieved, abduction strength will automatically be considered zero. To prevent leaning toward the contralateral side and the additional use of the trunk muscles, stabilization of the torso will be achieved by direct contact of the contralateral shoulder and trunk with a wall. Scapular rotation will be restricted by the examiner’s hand. Forearm supination will be tested with the elbow in 90° flexion, maximal pronation and 0° pronation/supination. Stabilization straps will be secured and foot markers noted during all testing to ensure a standardized and reproducible protocol. Three submaximal effort trials will be performed in each axis to acquaint the patient with the testing conditions. Three maximal effort trials will be performed in each axis and the recorded values will be averaged to measure the strengths. A 5-minute rest period will be allowed between each testing period.

The normal contralateral upper extremity will be used as a matched control in the evaluation of the postoperative biceps strength measurement without adjusting the results for handedness because no significant differences have been observed between the dominant and nondominant side of healthy volunteers [25]. Thus, the achieved average elbow flexion strength of the affected arm will be compared with the healthy opposite side of the patient, and the percentage will be graded between 0 and 20 points, as previously described [45]. In this regard, 20 points will be given to a performance of 91% and above; 16 points, between 90% and 81%; 12 points, between 80% and 71%; 8 points, between 70% and 61%; 4 points, between 60% and 51%; and 0 points to a performance below 50%.

The SF-36 is a validated, multi-purpose, short-form health survey with 36 questions, which combine eight health domains into a physical (PCS) and mental component scale (MCS) [46]. The PCS combines the health domains of physical functioning (PF), role limitations due to physical health (RP), bodily pain (BP), and general health perceptions (GH). The MCS combines the health domains of vitality, energy, or fatigue (VT), social functioning (SF), role limitations due to emotional problems (RE), and general mental health (MH). SF-36 scores range from zero to 100 points with lower scores indicating poorer function [47,48]. The postoperative difference in the SF-36 scores between the 2 groups will be evaluated at the two-year follow-up.

At the final follow-up, the patients and the blinded investigator will be asked to guess which treatment they believe the patient received to assess blinding and randomization.

### Complications and shoulder pain medication usage

The investigators who will be blinded to the surgical treatment will be responsible for identifying the complications related to the surgical procedure (e.g., the failure of tendon fixation, pain at the level of the bicipital groove, nerve injury, infection or skin scarring at the surgical site, cosmetic deformity) that might affect each subject throughout the study and follow-up period. Patients will be asked to provide baseline documentation on their medication usage for shoulder pain to be compared with the level of usage reported at the follow-up visits. The generic name, dosage, and frequency of these medications will be recorded. The presence of cramping will be noted following a verbal report from the patient.

### Statistical analysis

The mean, standard deviation and range will be reported for the continuous variables (symptom duration, CMS and SF-36 scores), whereas counts will be used to describe the categorical variables (gender, dominant arm, surgical side, concomitant injuries, disease onset, prior conservative therapies, shoulder tests, surgical technique, cramping, surgical complications, medication usage). Discrete variables will also be analyzed (activity level, LHBT condition). A determination of whether the CMS and SF-36 absolute and percent changes from baseline can reasonably be modeled using Gaussian theory will be performed by examining normal probability plots.

Mean CMS and SF-36 scores will be compared between and within treatment groups at each follow-up using repeated measure analysis of variance (ANOVA) methods on the appropriate scale. The chi-squared test will be used for the comparison of binary measures. If a parametric analysis is feasible, t-tests will be used for the comparison of continuous measures. If the Gaussian model is not appropriate, non-parametric comparison methods will be used on medians, as well as means. In particular, the Mann–Whitney U-test will be used to assess the difference in score distribution between the groups, whereas the Wilcoxon test will be used to compare the scores within the group. The percent of dropouts over time will also be compared in each treatment group. Complications will be compared by treatment group using exact chi-squared methods. Confidence intervals will be reported for the incidence of complications. Age-weighted univariate and multiple stepwise linear and logistic regression analyses will be used to evaluate the relationships between explanatory variables and outcomes with continuous and categorical distributions, respectively. Only explanatory and confounding variables that show a trend toward an association with the outcome of interest (e.g., p < 0.10) in the univariate analysis will be inserted into these models. In the multiple linear regression analysis, total adjusted R^2^ for the model and changes in R^2^ for the independent contribution of single predictors will be calculated to assess the total variance in the outcome variable accounted for by the whole model and single explanatory variables, respectively. In the case of collinearity between the explanatory variables, only the best-fitting models (e.g., those including the collinear explanatory variable accounting for the most relevant variation in the variance of the outcome) will be adopted. A P value of less than 0.05 will be considered significant. The SPSS (SPSS Statistics 17.0, Inc., Chicago, IL, USA) software program for Windows will be used for the database and statistical analysis.

### Ethics

The trial will be conducted in Fermo (Italy) and Catanzaro (Italy) according to the principles of the Helsinki declaration. Participants will receive information about the study protocol before providing written consent for inclusion in the study. Ethics approval for this study has been received from the Ethics Committee of the “Azienda Ospedaliera Mater Domini”, Catanzaro, Italy, (ref. number 2011–57), which acts as the central ethics committee for this trial. Approval has been obtained from the local ethics committee of the other participating center. With the patient’s permission, an informational letter describing the patient’s participation will be sent to his or her general practitioner. No special insurance will be provided for the patients because the protocol treatments are common surgical procedures routinely used in the centers involved in the study. All participant surgeons will be trained with regard to the study procedures at the beginning of the study.

## Discussion

To the best of our knowledge, this will be the first double-blind randomized controlled trial to compare clinical scores of tenodesis and tenotomy in the treatment of LHBT lesions. There are a few studies in the literature that directly compare the results of these techniques, and most of them reveal no differences between treatments [11,24-26,49,50]. Boileau *et al.* retrospectively evaluated 68 patients with irreparable rotator cuff tears treated with isolated arthroscopic biceps tenotomy or tenodesis and reported similar outcomes after both procedures [24]. Osbahr *et al.* retrospectively studied 160 patients with chronic refractive bicipital pain, treating half of the patients with tenotomy and the other half with tenodesis [49]. They found no significant difference between the two treatment methods in terms of cosmetic deformity, muscle spasm or anterior shoulder pain. Similarly, when twenty patients who underwent biceps tenotomy or tenodesis for chronic tenosynovitis were compared retrospectively, no difference in the functional or cosmetic results was observed [50].

Wittstein *et al.* showed no significant difference in postoperative scores between the two techniques in a cohort study of 35 patients [25]. The major limitations of this study were a lack of randomization of participants receiving tenotomy or tenodesis and a limited number of participants. In agreement with a retrospective study [11], the authors also found that tenotomy decreased forearm supination compared with tenodesis. These findings were not confirmed in other studies, which reported no difference between these techniques [16,24,26]. Shank *et al.* compared the forearm supination and elbow flexion strength of the upper extremity and found no significant difference between the tenotomy, tenodesis, and control groups [26]. However, they did not report the clinical scores after these surgeries [26]. Koh and colleagues demonstrated that neither clinical scores nor total surgical times revealed significant differences between tenotomy and tenodesis [16]. Notably, this study was not a randomized trial, and the study group was underpowered for most of the outcomes. Therefore, the authors acknowledge that their results should be interpreted with care.

The need for appropriately powered, well-conducted, randomized, controlled trials that compare the outcomes of these procedures has recently been emphasized [15]. To improve studies investigating biceps tenotomy versus tenodesis, the analysis of several variables, including patient demographics, concomitant injuries, CMS, surgical complications, and specific clinical outcomes, such as cramping or cosmetic deformity, has been recommended [14]. The results of our trial will provide important information to patients and health care providers about the effectiveness of these targeted interventions, possible complications, and predictors of outcome.

## Abbreviations

BP: bodily pain; CMS: Constant-Murley score; GH: general health perceptions; LHB: long head of the biceps; LHBT: long head of the biceps brachii tendon; MCS: Mental Component Scale; MH: general mental health; MRI: magnetic resonance imaging; NSAIDs: non-steroidal anti-inflammatory drugs; PCS: Physical Component Scale; PF: physical functioning; RE: role limitations due to emotional problems; RP: role limitations due to physical health; SF: social functioning; SF-36: short form 36; SLAP: superior labrum tear from anterior to posterior; VT: vitality energy, or fatigue.

## Competing interests

The authors declare that they have no competing interests.

## Authors’ contributions

OG and RC conceived this study and had full access to all data in the study. FF performed the statistical analyses. FF, GG, MD and OG took responsibility for the accuracy of the data analyses and wrote the manuscript. RC and GG performed a critical revision of the manuscript to verify important intellectual content. All authors read and approved the final manuscript.

## Pre-publication history

The pre-publication history for this paper can be accessed here:

http://www.biomedcentral.com/1471-2474/13/205/prepub
